# Parathyroid Hormone Related Protein (PTHrP)-Associated Molecular Signatures in Tissue Differentiation and Non-Tumoral Diseases

**DOI:** 10.3390/biology12070950

**Published:** 2023-07-03

**Authors:** Mariangela Librizzi, Flores Naselli, Giulia Abruscato, Claudio Luparello, Fabio Caradonna

**Affiliations:** Dipartimento di Scienze e Tecnologie Biologiche Chimiche e Farmaceutiche (STEBICEF), Università di Palermo, 90128 Palermo, Italy; librizzimariangela87@gmail.com (M.L.); flores.naselli@unipa.it (F.N.); fabio.caradonna@unipa.it (F.C.)

**Keywords:** cell biology, gene expression, adipose tissue, bone, cartilage, intestine, kidney, liver, lung, pancreas, skin

## Abstract

**Simple Summary:**

Parathyroid-hormone-related protein (PTHrP) is a protein hormone of 139, 141, or 173 amino acids, which may be cleaved into smaller bioactive forms, comprising amino terminus, mid-region, and carboxy terminus peptides, active as key controllers of viability, proliferation, and differentiation in diverse normal and pathological cell and tissue model systems via the reprogramming of gene expression and intracellular signalization. Within the large number of data available in the literature on this matter, the objective of this review is to review selected studies that report the detection of molecular markers of exposure to PTHrP, either as full-length protein or as discrete peptides, in peculiar normal or non-neoplastic biological contexts. In particular, the data presented relate to adipose, bone, dental, cartilaginous, and skin tissues, as well as intestinal, renal, hepatic, pulmonary, and pancreatic epithelia, with a focus on hepatic fibrosis-, pancreatitis-, and diabetes-related changes, as demonstrated by molecular profiling, briefly recapitulating the biological implications of the specific gene and/or pathway activation or inactivation.

**Abstract:**

Parathyroid-hormone-related protein (PTHrP) is encoded by the *PTHLH* gene which, via alternative promoter usage and splicing mechanisms, can give rise to at least three isoforms of 139, 141, and 173 amino acids with distinct C-terminals. PTHrP is subjected to different post-translational processing that generates smaller bioactive forms, comprising amino terminus, mid-region (containing a nuclear/nucleolar targeting signal), and carboxy terminus peptides. Both the full-length protein and the discrete peptides are key controllers of viability, proliferation, differentiation, and apoptosis in diverse normal and pathological biological systems via the reprogramming of gene expression and remodulation of PKA or PKC-mediated signalization mechanisms. The aim of this review is to pick up selected studies on PTHrP-associated signatures as revealed by molecular profiling assays, focusing on the available data about exemplary differentiating, differentiated, or nontumoral cell and tissue models. In particular, the data presented relate to adipose, bone, dental, cartilaginous, and skin tissues, as well as intestinal, renal, hepatic, pulmonary, and pancreatic epithelia, with a focus on hepatic fibrosis-, pancreatitis-, and diabetes-related changes as diseased states. When reported, the biochemical and/or physiological aspects associated with the specific molecular modulation of gene expression and signal transduction pathways in the target model systems under examination are also briefly described.

## 1. A Brief Note about Parathyroid Hormone-Related Protein (PTHrP) Structure and Function

PTHrP is the product of the *PTHLH* gene, which extends more than 15 kb of genomic DNA located on chromosome 12. The *PTHLH* gene exhibits a complex organization with three different promoters and alternative splicing mechanisms producing multiple mRNA variants which differentiate in their 3′ ends encompassing both coding and untranslated regions. Three isoforms of 139, 141, and 173 amino acids with distinct C-terminals are the protein products of the different translation patterns (Table 1). The extreme N-terminus displays sequence homology with PTH, thus binding with equal affinity to the shared G protein-linked PTH/PTHrP receptor PTH1R.

PTHrP isoforms are polyhormones subjected to different post-translational processing that generates smaller secretory forms of the peptide. These include PTHrP (1–36), which contains homology with PTH and activates PTH1R. Other peptides consist of the mid-region fragments, such as (38–94) and (67–86), which have been shown to influence transplacental calcium transport and the growth and invasive behavior of breast epithelial cells, and the C-terminal fragment comprising PTHrP (107–139), also known as osteostatin, which has been shown to act on skin, heart, and bone cells [1].

**Figure 1:**
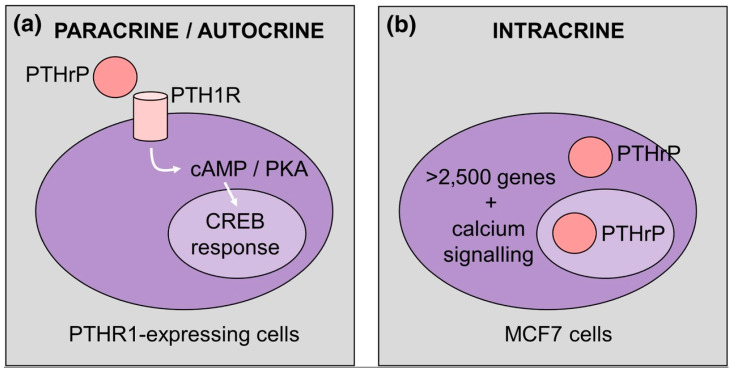
PTHrP regulation of gene expression via paracrine/autocrine (**a**) and intracrine (**b**) actions. (**a**) PTHrP-bound PTH1R stimulates cAMP/CREB signalization, whereas (**b**) gene expression is activated via alternative mechanisms, such as calcium signaling. Reprinted from [3].

The presence of a lysine/arginine-rich bipartite sequence in the mid-region PTHrP fragment, which is homologous to the nuclear/nucleolar targeting signal (NTS) present in SV40 large tumor antigen (able to direct importin β/Ran GTPase-mediated import), may allow an “intracrine” route supplementing the autocrine/paracrine counterpart (Figure 1) [3].

Over the past 30 years, a great deal of research has demonstrated that PTHrP participates in various complex signaling pathways through its membrane and nuclear effects. It has been shown that the full-length protein and its discrete fragments are multifaceted critical regulators of proliferation, differentiation, and apoptosis acting on PKA- or PKC-mediated signalization, whose main, but not exclusive, targets are P21, Akt, and NF-κB. A detailed molecular dissection of the involvement of PTHrP in signal transduction mechanisms has been the object of an extensive review literature [4,5]. On the other hand, PTHrP or its discrete domains have been proven to affect gene expression in a direct and substantial way in both normal, disease-affected, and neoplastic cells. A comprehensive recapitulation of PTHrP-dependent modulation of gene signatures in cancer cells has already appeared [6].

The aim of this review is to evaluate selected studies on PTHRP-associated signatures as revealed by gene expression profiling assays, focusing on the available data about exemplary differentiating, differentiated, or nontumoral cell and tissue models. When reported, the biochemical and/or physiological aspects associated with the specific molecular modulation in the target model systems under examination are also briefly described.

## 2. Adipose Tissue: PTHrP-Related Signatures in Adipogenesis and Transdifferentiation

Adipogenesis regulates adipose tissue expansion and function [7]. Despite the well-known role of PTHrP in the differentiation programming of stem cells into different cellular linages, its role in the regulation of adipogenic differentiation by human fat tissue-derived stem cells has only been partially elucidated at the gene expression level. Roca-Rodriguez et al. [8] showed that *PTHrP* was expressed in both visceral and subcutaneous adipose tissue in humans. Moreover, *PTHRP* expression progressively decreased during adipogenesis from undifferentiated mesenchymal cells. As a confirmation, data collected after *PTHRP* silencing in adipogenesis-committed stem cells demonstrated the downregulation of the adipogenic markers *PPARG2*, *FABP4*, *ADRP*, and *CEBPA*, coding for peroxisome proliferator activated receptor γ, fatty acid-binding protein 4, lipid droplet-associated adipose differentiation-related protein, and transcription factor CCAAT enhancer-binding protein α, respectively. Furthermore, they showed that *PTHRP* expression correlated with obesity-related morbidities, such as the setting of insulin resistance and the increase in body mass index and hip circumference in patients affected by type 2 diabetes, thereby defining PTHrP as a key regulator of their development. The mechanism via which PTHrP may switch the differentiation of stem cells from adipo- to osteogenesis, thereby inhibiting fat tissue formation, was studied in the rodent pluripotent mesenchymal cell line C3H10T^1⁄2^. Co-exposure of cells to bone morphogenetic protein-2 (BMP2) and PTHrP determined the downregulation of PPARγ and aP2 (adipocyte fatty acid-binding protein) and the concurrent upregulation of alkaline phosphatase, type I collagen, and osteocalcin mRNA levels. The PKC-mediated signalization was found to be at least in part implicated in this activity [9].

Dealing with bone-adipose tissue endocrine interplay, Zhang et al. [10] demonstrated that, in Ptch1^c/c^;HOC-Cre mutant mice, characterized by the perturbation of energy metabolism, the upregulated Hedgehog signalization increased bone-derived PTHrP release. This, in turn, triggered the modulation of the PKA/cAMP and Akt/Foxo pathways, leading to the overexpression of *UCP1*, coding for the uncoupling protein-1 that mediates energy expenditure via thermogenesis, with subsequent white adipose tissue (WAT) browning and enhancement of heat production. Moreover, PTHrP determined the increase of adiponectin mRNA and protein levels [10]. Adiponectin is involved in glucose homeostasis, because of its insulin-sensitizing activity [11,12], thereby indicating that PTHrP is also involved in the regulation of energy metabolism at this level. Since skeletal muscles also contribute to energy metabolism, it was also proven that in the mutant mice they underwent atrophy and adiponectin-triggered increase of fatty acid via significant upregulation of the genes whose products are involved in fatty acid oxidation (*ACO*, *CPT1*, and *FABP3*) and glucose uptake (*GLUT1* and *GLUT4*). The action of adiponectin was mediated by the activation of 5′-AMP-activated protein kinase (AMPK); moreover, in the mutant mice AMPK was also activated in the liver in which the expression of *GLUT1* was markedly increased. Therefore, in this experimental model, PTHrP, along with adiponectin’s contribution, was responsible for hypoglycemia due to glucose uptake and systemic fatty acid oxidation. As further support for this evidence, PTHrP was also found implicated in the higher rate of oxygen consumption and waste of fat and muscle tissues occurring in the Lewis lung carcinoma model of cancer cachexia developed in syngeneic C57BL/6 mice [13]. In particular, PTHrP (1–34), released among the tumor-derived factors, was proven to stimulate thermogenic gene expression, specifically upregulating *UCP1* and *DIO2*, the latter coding for type 2 iodothyronine deiodinase, a selenoenzyme which increases during cold stress only in brown adipose tissue, ultimately resulting in the onset of hypermetabolism [14].

A recent study by Qin and colleagues [15] highlighted the role of PTHrP in both opposing brown adipose tissue (BAT) whitening and promoting WAT browning in mice transduced with adeno-associated PTHrP-encoding virus vector, submitted to a high-fat diet (HFD) for some weeks. PTHrP was found to protect the animals from the diet–induced onset of obesity stimulating WAT transdifferentiation and maintenance of BAT via upregulation of *UCP1*, *UCP2*, and *PGC1α*, the latter coding for PPARγ coactivator 1α. Moreover, *VEGFA*, coding for vascular endothelial growth factor A, was upregulated and considered responsible of the concomitant state of inflammation in BAT. In parallel, in the liver of HFD-submitted mice, overexpression of PTHrP was proven not only to attenuate the transcription level of the genes coding for enzymes and receptors responsible of fatty acid synthesis (*ACSL1*, *FASN*, and *PPARG*) but also to enhance that of lipolysis-related enzymes and receptors (*ATGL*, *ACOX1*, CPT1A, and *PPARA*). In addition, *FGF21*, coding for fibroblast growth factor 21, an atypical member of FGF family active on glucose and lipid metabolism [16] and on adiponectin production, was prominently upregulated in the liver of PTHrP-overexpressing mice, thereby resulting beneficial for the mitigation of the obesity-linked metabolic diseases such as hepatic steatosis, insulin resistance, and glucose intolerance.

## 3. Bone and Dental Tissues: PTHrP-Related Signatures in Osteoblatogenesis, Osteoclastogenesis, and Ossification

The present paragraph reports selected examples referring to bone and dental tissues, since PTHrP-associated gene signatures in the bone remodeling process have been the object of extensive investigation since the late 1990s. One of the first pieces of evidence was provided by De Miguel et al. [17] who reported the PKC-mediated upregulation of IL-6 mRNA, a putative osteoblast differentiation factor also involved in bone resorption in various disorders such as malignant hypercalcemia and Paget’s disease, operated by PTHrP (1–34) and also (107–139) on osteoblasts from human trabecular bone (hOB). Both N- and C-terminal PTHrP were also proven to intervene in the production of VEGF by hOB cells and MG-63 osteosarcoma cells. Esbrit et al. [18] examined the underlying intracellular mechanism and demonstrated its induction at the transcriptional level and the implication of PKC signalization, thereby suggesting that the peptides may take part in the onset of vascularization in vivo during the endochondral ossification process. Additionally, Alonso et al. [19] produced interesting data on the potential anabolic action of the sole PTHrP (107–139) on bone mediated by its interplay with the VEGF system. In fact, the peptide was found to promote hOB and MG-63 cell survival by directly interacting with and transactivating VEGF receptor-2, as well as stimulating extracellular signal-regulated kinase (ERK) 1/2 and Akt signalization and RUNX2 activation. The anabolic action of the C-terminal peptide also aimed at tissue engineering applications was further described by Lozano et al. [20], who studied the effect of its introduction in gelatin–glutaraldehyde biopolymer-coated hydroxyapatite scaffolds aimed to improve the osteoinductive capability of this biomaterial for orthopedic implants. Using rat bone defect models and osteoblastic cell cultures, PTHrP (107–111) was found to downregulate *SOST*, *DKK1*, and *RANKL*, coding for the WNT pathway inhibitors sclerostin and Dickkopf-1 [21,22] and the osteoclastogenesis-inducing receptor activator of NF-κB ligand (RANKL), respectively, but to upregulate *VEGF*, *OCN*, and *OPG*, with the latter two coding for the osteoblast differentiation factors osteocalcin and osteoprotegerin [23], respectively. The formation of osseous trabeculae in the cavitary bone defects with osteoblasts adhering to the trabecular surface was observed, strongly suggesting that C-terminal PTHrP may restrain osteoclastogenesis during bone regeneration.

**Figure 2:**
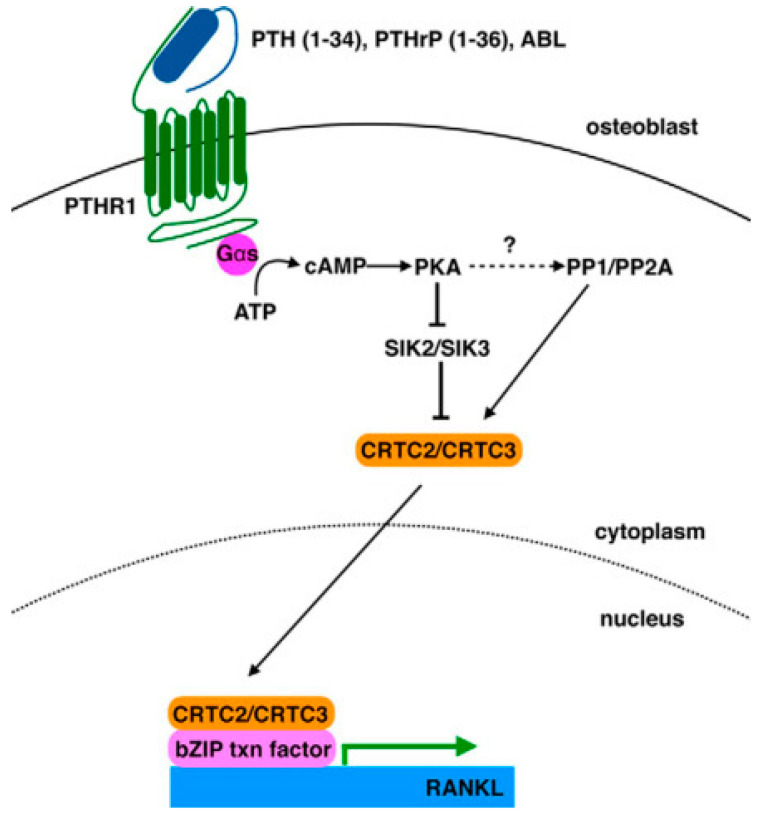
Model depicting the pathway induced by PTH (1–34), PTHrP (1–36), and abaloparatide (ABL), which leads to *RANKL* upregulation. Reprinted from [27]. Distributed under the terms of the Creative Commons Attribution licence (CC BY 4.0).

In addition to the paper commented on above, other articles highlighted the effect of PTHrP on the modulation of RANKL and OPG mRNA transcription. The periodontal ligament (PDL) plays an important role in root resorption of human deciduous teeth by odontoclasts. To assess how PDL cells are involved in osteoclastogenesis regulation, Fukushima et al. [24] examined the effects exerted by factors secreted by the tooth germ, including PTHrP. N-terminal PTHrP was found to induce osteoclast differentiation via the induction of *RANKL* and the reduction in *OPG* expression in PDL cells with partial contribution of the PKC, but not PKA, pathway. Additionally, Sun et al. [25] reported the ability of PTHrP to upregulate *RANKL* and downregulate *OPG* in human dental follicle cells, thereby influencing ostoclastogenesis. Data from Mak et al. [26] demonstrated that *PTHRP* expression in mature osteoblasts was under the control of Hedgehog signaling. Ricarte et al. [27] analyzed the effects of PTH (1–34), PTHrP (1–36), and N-terminal PTHrP analog abaloparatide, i.e., [Glu^22,25^, Leu^23,28,31^, Aib^29^, Lys^26,30^] PTHrP (1–34)–NH_2_, on *RANKL* expression by osteoblasts and unveiled some molecular aspects of the regulation, being based upon the induction of a particular arm of the cAMP/PKA/salt-inducible kinase (SIK) signaling axis, and involving the nuclear localization of CREB-regulated transcription coactivator (CRTC)-2 and -3 mediated by the Ser/Thr phosphatases PP1 and PP2A (Figure 2).

Interestingly, in the study by Elango et al. [28] on the osteodifferentiation of mesenchymal stem cells (MSCs), the cooperative inhibitory effect of PTHrP and soluble RANKL (sRANKL), a circulating form released in vivo by disintegrin metalloproteinase-mediated cleavage of the membrane-bound component, was demonstrated. The molecular signatures associated with the co-exposure-mediated osteogenesis inhibition included the enhanced downregulation of OPG, OCN, collagen, and cellular alkaline phosphatase mRNA transcription coupled with the decreased levels of mineral deposition conceivably due to the inhibition of the progressive ankylosis protein (ANK) signaling pathway, whose deficiency is known to impair osteoblastogenesis and bone formation [29]. The mineralization inhibition of bone nodules, coupled with the downregulation of bone sialoprotein mRNA and protein levels, was also found by Kamel and Yee [30] after continuous and intermittent exposure of primary rat calvarial cells to N-terminal PTHrP. Wang et al. [31] demonstrated that administration of PTHrP was able to redress impaired bone fracture healings in PTHrP-deficient osteoporotic mice, as also revealed by the increase in bone formation-related gene and protein expression levels, i.e., those of alkaline phosphatase, type I collagen, RUNX2, and insulin-like growth factor (IGF)-1.

**Figure 3:**
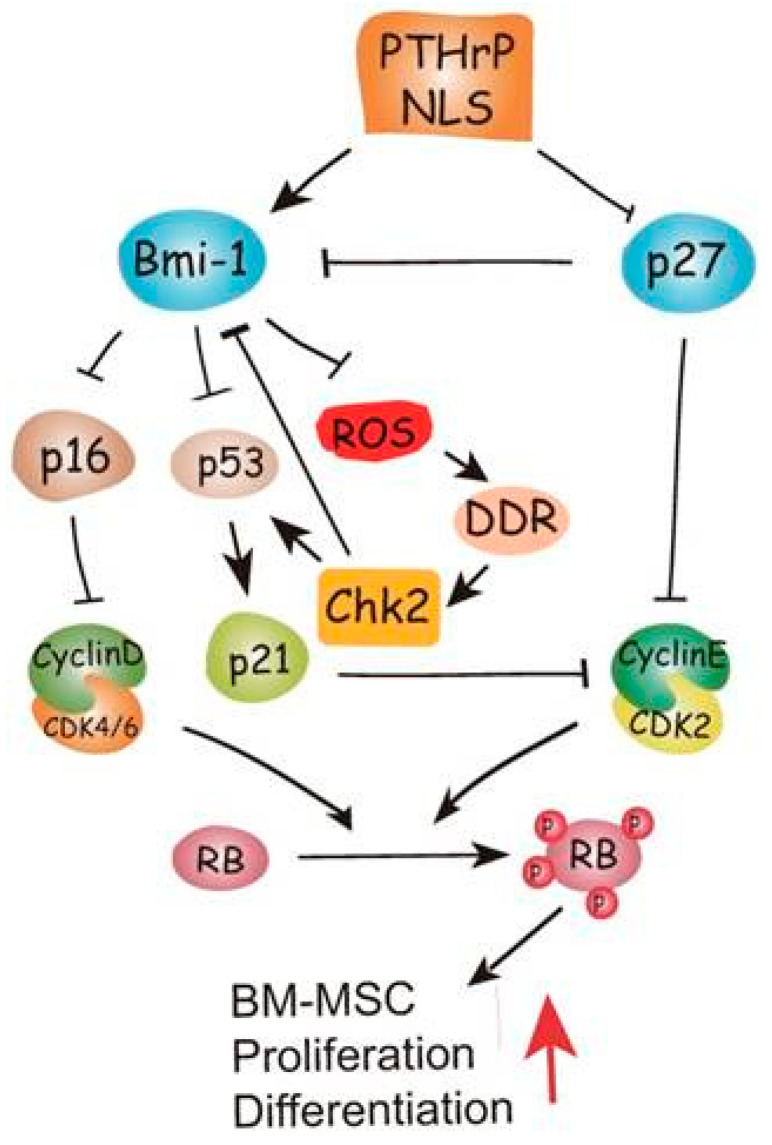
Model depicting the pathways modulated by the PTHrP NTS (here referred to as NLS, i.e., nuclear localization sequence) and C-terminus, stimulating osteogenesis by bone marrow MSC. Reprinted from [33]. Distributed under the terms of the Creative Commons Attribution (CC BY-NC) license.

Zhu et al. [32] elucidated the mechanism via which the PTHrP NTS and C-terminal fragment promote bone formation using mutant mice expressing PTHrP (1–84), a truncated form lacking both regions and homozygous for p27^KIP1^ deletion, examining whether the latter protein might function downstream of the PTHrP domains. Using the same animal model, Zhang et al. [33] examined whether the growth arrest and senescence generated in the mutant mice might be associated with oxidative stress and DNA damage response by assessing the effects of deletion of the checkpoint kinase-2 (CHK2) regulator. As shown in Figure 3, their cumulative results indicated that PTHrP NTS and C-terminus inhibited p27^KIP1^, thus stimulating Bmi-1 protein, which, in turn, inhibited p16 and p53. This inhibitory circuit allowed the cyclin D/CDK4/6 and cyclin E/CDK2 complexes to trigger the progression of bone marrow MSCs along the osteoblastic lineage via Rb phosphorylation. Moreover, activated Bmi-1 downregulated ROS levels and suppressed the activation of the DNA damage response pathway that plays a possible role as a downstream target in the action of PTHrP to regulate skeletal development and growth.

**Figure 4:**
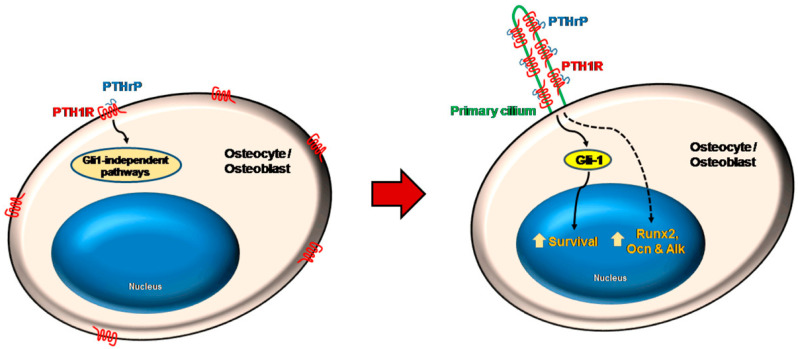
Model depicting the pathways activated by PTHrP and inducing survival- and bone formation-related gene expression by mouse osteoblasts and osteocytes. Primary cilia- and Gli-1-dependent mechanisms are involved in the pro-survival action, whereas primary cilia-dependent and Gli-1-independent pathways mediate the overexpression of the osteogenic genes *Runx2*, osteocalcin (*Ocn*), and bone alkaline phosphatase (*Alk*). Reprinted from [34].

Noteworthily, Martin-Guerrero et al. [34] reported for the first time the localization of PTH1R in bone cells’ primary cilia and proposed that PTHrP’s pro-survival action, likely following the mechanical stress-promoted transport of PTH1R to the appendage surface, may be mediated by the upregulation of the Hedgehog effector GLI family zinc finger 1 (Gli1) protein, along with the subsequent overexpression of Hedgehog transcription factor and activation of the related signaling pathway. On the other hand, PTHrP osteogenic activities, such as RUNX2, OCN, and OPG upregulation, appeared to be mediated by primary cilia-dependent, but Gli1-independent, mechanisms (Figure 4).

Regarding the influence of PTHrP on senescence features led by inflammatory diseases, such as osteoarthritis (OA), in osteoblasts, the study by Platas et al. [35] showed that the C-terminal peptides PTHrP (107–111) and (107–139), but not the N-terminal domain, were able to reduce remarkably the expression of senescence markers induced in vitro by treatment of OA osteoblasts with IL-1β. In particular, the molecular signatures associated with the exposure to the peptides were the downregulation of p53, p21, p16, COX2, caveolin 1, and AP-1 transcription factor-coding genes. In addition, the decreases in the activation of NF-κB, accumulation of γH2AX (histone marker of inflammation-induced DNA damage and aging), and production of PGE_2_ and IL-6, and the promotion of matrix mineralization were also reported, thereby supporting the hypothesis of the beneficial antisenescence and anti-inflammatory action of the C-terminal moiety of PTHrP.

PTHrP also protects osteoblasts from oxidative stress, one of the several factors that prompt their apoptosis. In particular, Ardura et al. [36] demonstrated that PTHrP (1–37) could prevent the H_2_O_2_-induced p38 and ERK phosphorylation in osteoblastic MC3T3-E1 and MG-63 cells, as well as the high oxidative stress in an animal model, by increasing the mRNA expression of catalase and MAPK phosphatase-1. This suggested that the antiapoptotic role of N-terminal peptide was accomplished by both reversing MAPK phosphorylation triggered by ROS and decreasing ROS levels.

In bone physiology, the cysteine–X–cysteine (CXC) family chemokine ligand 1 (CXCL1) is an important neutrophil chemoattractant acting during angiogenesis and inflammation. Interestingly, Onan et al. [37] identified its upregulation as a PTHrP (1–141)-associated signature in osteoblastogenesis and suggested that the chemokine may play a fundamental role in attracting the osteoclast precursors, which expose its receptor CXCR2 on their surface to the bone environment at the modeling sites.

Another example of molecular interactors associated with PTHrP, which does not exhaust the lengthy list present in the literature, is represented by the components of the WNT pathway. In this regard, a number of data were obtained in type I diabetes mellitus (TIDB) mouse models in which the inactivation of the WNT pathway and a decrease of bone mass occur, markedly augmenting fracture risk. Portal-Núñez et al. [38] reported that administration of PTHrP (1–36) and (107–139) resulted in the stabilization of β-catenin mediated by the downregulation of CSNK1A1, coding for the casein kinase I isoform α which is involved in the phosphorylation and degradation of catenin, together with GSK-3β. In addition, the sole N-terminal peptide was able to reverse the decrease in Wisp-1, a final component of the pathway acting on growth promotion, whereas the sole C-terminal peptide increased the expression of WNT11, a noncanonical WNT pathway activator. Moreover, as also demonstrated by Maycas et al. [39], in the diabetic mice, the peptides reversed the upregulation of SOST, coding for the WNT-β catenin inhibitor sclerostin, thus contributing also in this way to bone formation. Noteworthy, the N-terminal PTHrP analog abaloparatide was also proven to suppress, in addition to SOST expression, that of DKK1, coding for the WNT signaling antagonist Dickkopf-related protein 1 [40].

**Figure 5:**
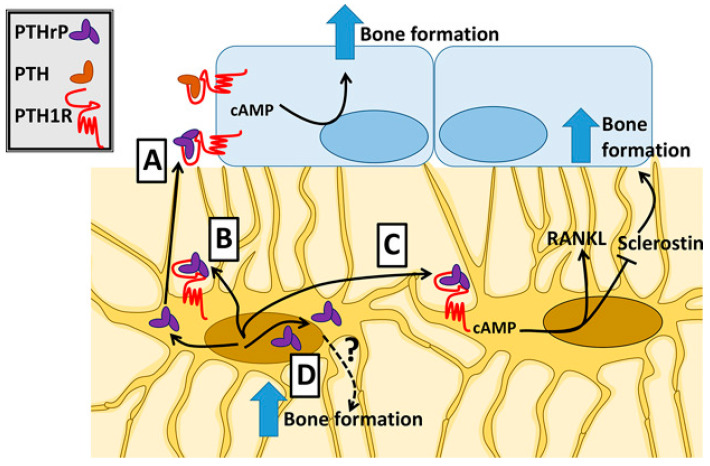
Model depicting the mechanisms of action of osteocyte-derived PTHrP. Full-length PTHrP is secreted in the lacunocanalicular network (A) and can activate autocrine (B) and paracrine pathways (C) via PTHR1–cAMP signaling resulting in the modification of gene expression. In addition, NTS-containing PTHrP fragments can also stimulate osteogenesis through a poorly elucidated intracrine action (D). Reprinted from [41].

Interestingly, Ansari et al. [41] demonstrated the occurrence of both autocrine/paracrine and intracrine mechanisms of action by osteocyte-produced PTHrP, depicted in Figure 5. In the former case, the full-length protein is released in the extracellular milieu and flows along the lacunocanalicular network, thus acting on the neighboring bone cells to promote osteogenesis via PTHR1/cAMP signaling-mediated modification of gene expression, such as the downregulation of sclerostin and the upregulation of RANKL, which is, however, insufficient to support osteoclastogenesis. In the latter case, bone formation and massive ECM production are stimulated by a non-receptor-mediated activity likely promoted at the nuclear and/or cytoplasmic level by NTS-containing mid-region peptides. Similarly, Pieles et al. [42] reported the occurrence of both mechanisms of action by dental stem cell-produced PTHrP, with alkaline phosphatase production being a result of its intracrine activity in addition to the receptor-mediated autocrine/paracrine action responsible for the differentiation of stem cells toward cementoblasts of the acellular cementum, periodontal ligament cells, and alveolar cryptal bone osteoblasts, as demonstrated by Takahashi et al. [43].

## 4. Cartilaginous Tissue: PTHrP-Related Signatures in the Repression of Chondrocyte Differentiation

Several early lines of evidence have revealed that PTHrP opposes chondrogenic differentiation both in vitro and in vivo [44,45,46]. Ito et al. [47] reported that, in mouse chondrogenic embryonic carcinoma ATDC5 cells, PTHrP negatively modulated the mRNA levels of bone morphogenetic protein 4 (BMP4), thus decreasing the amplitude of the chondrogenic signal. Moreover, in chick upper sternal chondrocytes cotreated with BMP2, PTHrP abrogated the expression of RUNX2, which, in the cartilage acts, as an inducer of chondrocyte maturation both in vitro and in vivo [48]. Interestingly, subsequent data indicated that PTHrP repressed chondrocyte hypertrophy via the PKA-activated dephosphorylation of histone deacetylase 4 (HDAC4) [49], thus adding epigenetic regulation as a possible parallel further mechanism of action in PTHrP-blocked chondrogenesis.

In antler chondrocytes, PTHrP increased the expression of *BCL2*, whose product is involved in the suppression of chondrocyte apoptosis, and *CCND1*, coding for cyclin D1, while downregulating *RUNX2*, thereby promoting cell proliferation while preventing differentiation [50]. In addition, in the same cell model system PTHrP was found able to downregulate *MMP13* and *MMP9*, whose products are metalloprotease 13 (collagenase-3) and −9 (92 kDa type IV collagenase), thereby restraining the proteolysis and remodeling of cartilage extracellular matrix (ECM), and blocking its maturation [51].

Particularly peculiar are the data obtained in normal articular human chondrocytes led to hypoxia. In fact, *PTHRP* was reported to be upregulated by oxygen deprivation, acting, in turn, as a positive regulator of the key cartilage transcription factor SOX9 [SRY (sex determining region on the Y chromosome)-box 9], which led to the increased expression of *COL2A1*, coding for the α_1_ chain of type II collagen [52]. Hypoxia-exposed MSCs showed the PTHrP-stimulated downregulation of *MEF2C*, coding for the transcriptional factor myocyte enhancement factor 2C. This suppressed chondrocytes’ hypertrophy by reducing the expression of *COL10A1*, coding for the α_1_ chain of type X collagen which is released by hypertrophic chondrocytes during endochondral osteogenesis [53].

Chondrogenesis was also studied in the case of a pulse, intermittent PTHrP treatment on MSCs. Noteworthily, the results obtained demonstrated that, while the constant application of PTHrP (1–34) suppressed chondrogenesis, conversely, pulsed exposure acted as a stimulator, as shown by the upregulation of *COL2A1* and reduced undesired hypertrophy, thus representing a best novelty in clinical treatments of cartilage defect regeneration [54].

Furthermore, animal models in vivo showed the crucial importance of PTHrP and associated pathways in modulating chondrogenesis. From the early work of Karaplis and colleagues [55], it was found that knockout mice for *PTHRP* die at birth with extensive chondrocyte hypertrophy. More than 20 years after, using multiple mouse genetic models Nishmori et al. [56] demonstrated that class I and II histone deacetylases (HDACs) are necessary for PTHrP action on chondrocyte differentiation. In particular, HDAC4 as the first protagonist and HDAC5 as an additional mediator were both involved in PTHrP signaling in chondrocytes. PTHrP action on HDAC4 appeared to suppress myocyte enhancer factor 2 (Mef2) function, thereby allowing the expression of *RUNX2*, necessary for chondrocyte hypertrophy. In consideration of the epigenetic role of the HDACs, these features open new scenarios at the second level for the PTHrP-mediated gene expression regulation in chondrocyte differentiation.

## 5. Intestinal Epithelium: PTHrP-Related Signatures in Calcium Uptake by Enterocytes

Since PTHrP was initially identified as a tumor-derived PTH-resembling hypercalcemic factor, early studies were focused on elucidating its potential role in calcium transport using normal chick intestine as a model system. Indeed, the N-terminal PTHrP (1–40) domain was proven to stimulate the rapid Ca^++^ uptake in the perfused chick duodenum via a signal transduction pathway in which Ca^++^ channels are activated [57]. In mammals, a complex homeostatic system regulates the extracellular calcium concentrations, particularly calcium uptake in the small intestine, conceivably through paracellular or transcellular transport. Paracellular calcium transport is concentration gradient-dependent, thereby representing passive transport. On the other hand, transcellular transport is an active, energy-dependent transport; consequently, it is highly regulated. The three steps of the transcellular calcium transport include calcium entry at the apical membranes through the epithelial calcium channel, the transport from the apical membrane to the basolateral membranes mediated by calcium-binding proteins, and the release of calcium from basolateral membrane via calcium transporters [58,59]. Liu et al. [60] investigated the molecular mechanism via which PTHrP induced calcium uptake in rat enterocytes and demonstrated that only PTHrP (1–40), but not (67–86) and (134–143), was able to induce a rapid calcium internalization. At the gene expression level, this was paralleled by the upregulation of *TRPV6*, *CALB1*, *NCX1*, and *PMCA1*, coding for potential vanilloid member 6 (a transcellular calcium transporter protein), calbindin, sodium–calcium exchanger 1, and plasma membrane calcium ATPase 1, respectively. Noteworthily, the first two proteins are known to be necessary for calcium uptake, whereas the other two are responsible for calcium extrusion from the basolateral membrane and, therefore, not involved in calcium internalization procedures. Furthermore, PTHrP (1–40) treatment increased PTHR1 mRNA level, as well as PKCα/β and PKA protein levels. Calcium uptake was inhibited when rat enterocytes were pretreated with antibodies or inhibitors of either calcium transmembrane transporters, PTHR1 receptors, or PKCα/β, thus demonstrating that the intracellular pathway mediated by the last signal transducer was that involved in PTHrP-induced calcium resorption.

Furthermore, the PKA/RUNX2 pathway appeared to be involved in PTHrP-triggered epithelial–mesenchymal transition (EMT) in intestinal epithelial cells. In particular, the PTHrP/PTHR1 system was found to play an important role in the onset of EMT that promotes intestinal epithelial cell transition into fibroblasts, thus inducing intestinal fibrosis. He et al. [61] demonstrated that the PTHrP/PTHR1 system induced *RUNX2* expression and subsequent deposition of type I collagen via PKA pathway activation in an animal model of intestinal fibrosis and in the intestine of Crohn disease patients. Indeed, the PTHrP-triggered stimulation of EMT-related markers and type I collagen accumulation was blocked when *RUNX2* was genetically silenced with siRNA demonstrating that the PKA–RUNX2 axis was implicated in the process. Taken together, these data show that PTHrP plays a pivotal role in the establishment of the fibrotic disease.

## 6. Liver Parenchyma: PTHrP-Related Signatures in the Fibrotic Reaction of Hepatic Stellate Cells

Hepatic stellate cells, which reside in the subendothelial area of the Disse space, are triggered to myofibroblast-like cell differentiation by factors released by the damaged hepatocytes and are responsible of the remodeling of the ECM and the fibrotic reaction associated with the onset of chronic liver injury [62]. Liang et al. [63] reported that administration of PTHrP (1–36) to human normal hepatic stellate cells and the LX-2 cell line in vitro increased the mRNA and protein levels of α smooth muscle actin (α-SMA; myofibroblast marker), collagen I (principal constituent of the ECM), MMP-2 (contributing to the alteration of normal liver ECM [64]), and TGF-β1 (main cytokine involved in fibrosis). Additional experiments on the CCl_4_-induced mice model of hepatic fibrosis demonstrated that the PTHrP mRNA levels increased in a time-dependent manner; in this case, they were also related to the upregulation of the genes coding for TGF-β1, collagen I, and α-SMA. The same result was achieved by overexpressing PTHrP via administration of a liver-targeted PTHrP recombinant adeno-associated vector through the mice’s tail vein, and the animals developed spontaneously liver fibrosis within 6 months. Moreover, it was observed that, in PTHrP-induced fibrosis, the expressions levels of *PTCH*, *SHH* and *GLI2* genes, coding for Patched-1, Sonic Hedgehog, and GLI Family Zinc Finger-2 proteins, respectively, were also increased, indicating that PTHrP’s effect on the activation of stellate cells was mediated through Hedgehog signalization by the PTH1R/PKCθ/Hedgehog axis [65]. These cumulative results strongly suggest that PTHrP might be considered as one of the cytokines playing a central role in the onset of liver fibrotic disease.

## 7. Lung Epithelium: PTHrP-Related Signatures in Pulmonary Surfactant Production

In the lung, the PTHrP-associated gene regulatory network is involved in the control of fundamental aspects of the development and homeostasis of the organ, such as the epithelial–mesenchymal paracrine crosstalk and the production of surfactant [66]. In their study aimed at understanding the mechanism of these events during the differentiation process of epithelial type II (TII) cells, Torday and Rehan [67] showed that PTHrP acted as a “stretch-sensitive” product of such cells. In particular, stretching stimuli induced pulmonary surfactant production through a paracrine epithelial–mesenchymal–epithelial loop also mediated by leptin, a soluble product of the mature lipofibroblast (LFs). Their data demonstrated that stretching of LFs and TIIs in coculture resulted in the enhancement of PTHrP signaling from the epithelium to the mesenchyme through the coordinated upregulation of both *PTHRP* itself and *PTH1R* expression by TFII cells and LFs, respectively. In turn, stretching increased the signaling from the mesenchyme to the epithelium by augmenting leptin stimulation of the surfactant’s phospholipid synthesis by TII cells, which could be blocked by inhibitors of PTHrP or leptin, thus supporting the existence of the paracrine feedback loop. A PTHrP-related signature identified in this cAMP/protein kinase A-dependent mechanism was the upregulation of the mRNA of adipocyte differentiation-related protein (ADRP), involved in the mechanism of lipid uptake by LFs [68]. As further confirmation of the leptin/PTHrP axis, Oruqaj et al. [69] showed that elevated plasma leptin levels induced in high fat diet-fed mice upregulated the pulmonary mRNA expression of PTHrP, PTH1R, and other classical PTHrP downstream target genes such as *ADRP* or the key lipogenic marker *PPARG*; conversely, genetically leptin-deficient mice displayed reduced steady-state levels of PTHrP. Interestingly, PTHrP was also proven to exert an inhibitory effect on the transdifferentiation of LFs into myofibroblasts, commonly associated with nicotine exposure, via PPARγ upregulation [70]. Thus, the sustained activation of the PTHrP-driven epithelial–mesenchymal paracrine crosstalk may also act as a protective mechanism for prevention of the nicotine-induced lung injury.

## 8. Exocrine and Endocrine Pancreas: PTHrP-Related Signatures in β-Cell Biology and in the Onset of Pancreatitis

Sawada et al. [71] examined the effect of the differentiation status of pancreatic insulin-producing cells on PTHrP synthesis using different passages of the MIN6 cell line as an in vitro model system of well-differentiated and less-differentiated β cells. PTHrP was found expressed and secreted in both conditions, but increased with increasing passage, i.e., moving toward a less-differentiated phenotype. Moreover, the expression and secretion of furin, the PTHrP precursor-processing enzyme [72], followed the same trend, whereas *PTH1R* expression was similar in all the experimental conditions tested. Furthermore, PTHrP increased the insulin mRNA content through the cAMP pathway in highly differentiated MIN6 cells, as well as in primary cultured islets; on the other hand, it stimulated DNA synthesis, a marker of cell proliferation, in the less-differentiated MIN61 cultures. These data strongly suggested that PTHrP might be a regulator of the balance between growing and differentiated cells in the heterogeneous β-cell population of pancreatic islets. Within this context, PTHrP responded to an insulin shortage by inducing cell growth in the proliferation-prone cell fraction in an autocrine manner and induced insulin upregulation in highly differentiated cells, thus compensating for a loss in insulin-producing capacity in the whole cell mass in a paracrine manner.

Studies by Guthalu Kondegowda et al. [73] showed that PTHrP (1–36) was sufficient to exert biological effects on β-cell proliferation and function in vitro. The molecular signatures associated with cell growth following peptide administration were the late G_1_/S cell cycle activators cyclin E and cdk2, overexpressed in treated human islets, which caused an increase in the percentage of cells in the S-phase of the cell cycle acting in a synergistic way. This was coupled with a significant enhancement of insulin secretion at both physiological and increased glucose concentrations, thus confirming that exposure to PTHrP did not lead to cell dedifferentiation. Noteworthily, different results were obtained by analyzing the islets from rat insulin II promoter (RIP)-*PTHRP* transgenic mice [74] since the difference in cell cycle-related gene expression levels exists in the upregulation of the G_1_/S activator cyclin D2 and the downregulation of the inhibitor p16Ink4a, thereby suggesting that the systemic and acute administration of the sole N-terminal peptide to adult mice may stimulate β cell replication via different mechanisms.

**Figure 6:**
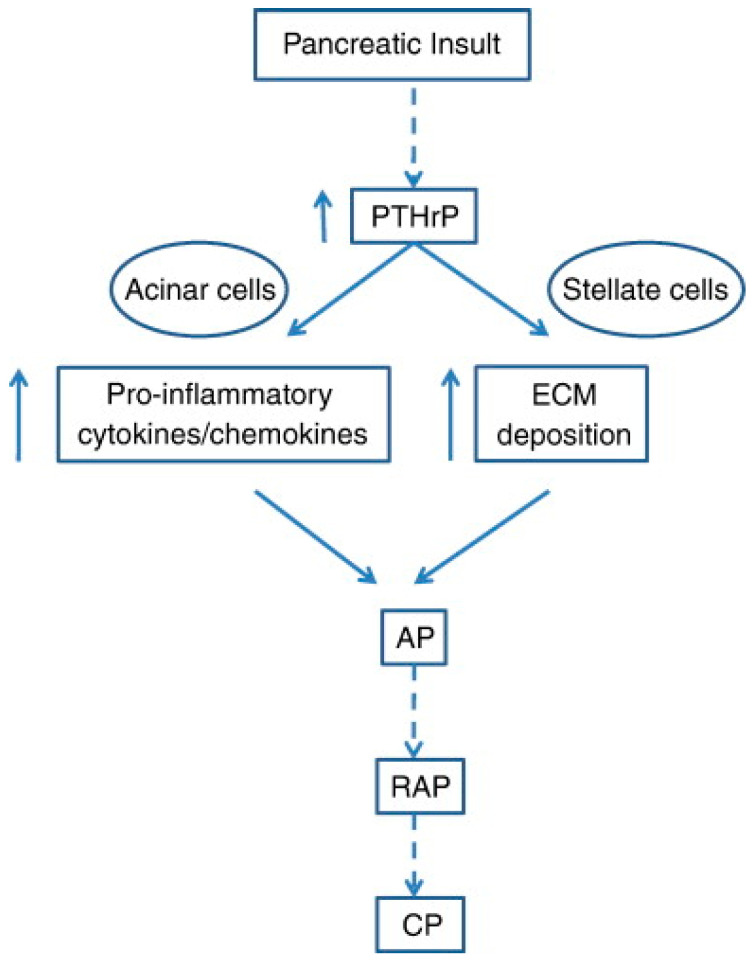
Scheme depicting the pathways activated by PTHrP, which induce the release of cytokines and chemokines and the deposition of the extracellular matrix (ECM). Activation of the stellate cells exacerbates the inflammatory and fibrogenic responses that accompany acute pancreatitis (AP), whose repeated episodes (RAP) may eventually lead to chronic pancreatitis (CP). Reprinted from [75].

In addition to the effect exerted by PTHrP in normal physiology, its involvement in pancreatic diseases was studied. Within this context, the studies by Bhatia at al. [75] focused on the molecular framework associated to the onset of acute pancreatitis, a common and potentially lethal necro-inflammatory disease of the exocrine pancreas that can degenerate into a chronic form with an elevated risk of cancer development [76]. Indeed, treatment of acinar and stellate cells, from both primary and stabilized (AR42J and irPSCc3) cultures with cerulein or ethanol induced an increase in PTHrP at mRNA and protein level. The molecular signatures associated with acinar cell exposure were the upregulation of the mRNA levels for IL-6 and ICAM-1, both factors involved in the control of the inflammatory response, whereas those associated with stellate cell exposure were the upregulation of the mRNA levels for fibronectin and procollagen type I, which are key mediators for the development of fibrosis. In addition, the treatment with PTHrP addressed acinar cells to apoptosis. Conceivably, the increase in PTHrP levels following alcohol abuse may be the starting point of a cascade of events that ultimately leads to the inflammatory and fibrotic response and to the loss in acinar cells due to apoptotic promotion. The pathway proposed by the authors for the contribution of PTHrP to the development of acute pancreatitis is shown in Figure 6.

The complex PTHrP-involving molecular network occurring in chronic pancreatitis was investigated by Rastellini et al. [77]. It is known that BMP2, apelin, and PTHrP signaling systems are expressed in the rodent and human pancreas, and that TGF-β is a major effector in the genesis and progression of the disease [78,79,80]. They found a bidirectional, negative feedback loop that targeted the transcriptional regulation of apelin and PTHrP; in particular, the anti-inflammatory and antifibrotic role of BMP2 appeared to be mediated by the concurrent activation of apelin and inhibition of PTHrPsignaling during pancreatitis. In addition, TGF-β upregulated PTHrP expression; in turn, both PTHrP and TGF-β stimulated the expression of gremlin10, an inhibitor of the BMP2/apelin axis that regulates the downstream inflammatory response and fibrosis. On the other hand, BMP2 and apelin inhibited PTHrP expression, thereby quenching the inducing effects of PTHrP, and possibly TGF-β, on inflammation and fibrosis. Therefore, these cumulative results substantiated the occurrence of a composite interplay underlying the response to the chronic injury that can either block or amplify organ deterioration associated with the disease, and that may offer insights for pharmacologic interventions for the treatment of this pathology.

## 9. Renal Parenchyma: PTHrP-Related Signatures in the Promotion of Kidney Cell Survival and in Diabetes-Related Changes

Within the context of the investigation on the pro-survival role of PTHrP in cells subjected to apoptotic stimuli, studies by Okoumsassoun et al. [81] on human Hek293 embryonic kidney cells addressed to apoptosis during treatment with TNFα showed that the expression of full-length PTHrP or the pre-exposure to its NTS peptide blocked the onset of programmed cell death as evidenced by the lack of caspase activation and the upregulation of antiapoptotic proteins at the mitochondrial membrane. This was accomplished through the increase in mRNA expression of casein kinase 2 (CK2), a known potent suppressor of apoptosis [82], followed by its sustained accumulation and rapid co-translocation with PTHrP to the nuclear compartment. In addition, albeit homogeneously present in the mitochondria regardless of the presence of PTHrP, CK2 activity was significantly increased in cells expressing PTHrP or pretreated with the NTS peptide. A link between PTHrP and CK2 in mediating nuclear and mitochondrial events related to cell survival was postulated on the basis of the possible phosphorylation activity of the kinase on PTHrP which may modify its RNA-binding activity, as well as CK2 targeting of the caspase 8 repressor protein ARC and the mitochondria-associated proteins Bid and Bad. Moreover, NTS exposure reduced the expression levels of *BAK*, *BAD*, and *BID* genes, thereby suggesting also an intervention at the transcription level.

**Figure 7:**
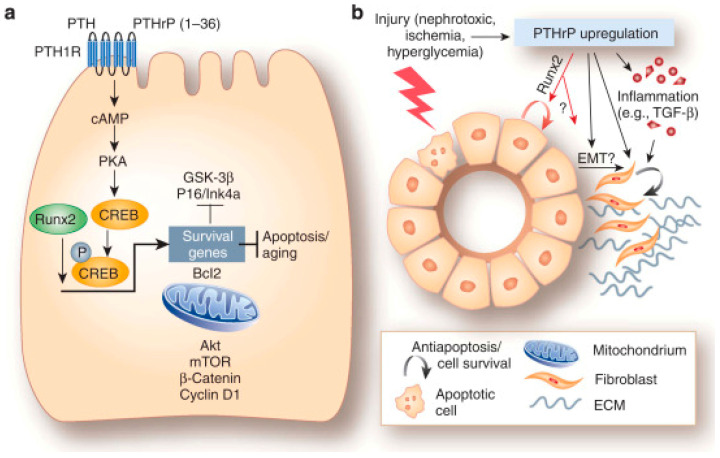
(**a**) Model summarizing the effect of PTHrP (1–36) in tubuloepithelial cell survival and antiapoptotic mechanisms. (**b**) Role played by the upregulation of PTHrP following kidney injury. A complete explanation of the pathways depicted can be found in the original paper [86].

It Is known that, after renal injury, the EMT process is committed to the rescue of the damaged tubules. In fact, the surviving tubular epithelial cells dedifferentiate into mesenchymal cells that migrate toward the damaged areas and subsequently redifferentiate into the original phenotype to restore tubular integrity. TGF-β is a key regulator of EMT, and PTHrP (1–36) was also proven to intervene in modulating the process. In particular, Ardura et al. [83] showed that the peptide upregulated TGF-β mRNA and protein in mouse tubular cells and that, in turn, antagonizing TGF-β strongly diminished EMT induction by N-terminal PTHrP. This suggested that TGF-β was a downstream mediator of PTHrP action, which occurs via EGFR activation of ERK1/2 pathway known to interfere with the integrity of intercellular junctions and to induce the accumulation of cytoplasmic β-catenin, both events linked to EMT onset [84]. These first data were subsequently supplemented by evidence demonstrating that the pro-survival action of PTHrP (1–36) was also mediated by the over-expression and increased nuclear translocation of RUNX2, instrumental for the up-regulation of the anti-apoptotic proteins Bcl-2 and osteopontin in mouse and human tubuloepithelial cell lines and in PTHrP-overexpressing transgenic mice in vivo [85]. A schematic model for the mechanism of N-terminal PTHrP pro-survival and antiapoptotic activities in tubuloepithelial cells was proposed by Kramann and Schneider [86] (Figure 7).

The viability-promoting role of PTHrP was confirmed by Hochane et al. [87] using primary cultures of murine mesangial cells (MC) in an in vitro model of inflammatory response mimicking the onset of mesangial proliferative glomerulonephritis. Exposure to IL1-β and TNFα was proven to upregulate *PTHRP* expression and extend the half-life of its mRNA through binding with HuR, an RNA-stabilizing factor known to interact with the 3′-UTR of the PTHrP-141 mRNA isoform, and delaying its degradation [88]. In addition, PTHrP treatment on MC cells induced an upregulation of a panel of cytokines and chemokines, as well as of IL1-β itself, suggesting a positive feedback loop on this gene expression. Furthermore, PTHrP treatment upregulated *COX2*, a target gene of NF-κB [89], and this overexpression was blocked by an inhibitor of IκB kinase, indicating the PTHrP-mediated activation of NF-kB pathway in MC. Interestingly, the upregulation of *COX2* was able to prevent apoptosis through the cAMP/PKA and PI3K/Akt pathways. Thus, Hochane and coworkers proposed a mechanism in which PTHrP is overexpressed after the exposition of MC to IL1-β and TNFα proinflammatory cytokines and acts itself as an inflammatory cytokine, increasing the expression levels of interleukins, chemokines, and COX2 through the NF-κB pathways, while also acting as a survival factor through the reduction in the apoptotic effects of IL1-β and TNFα via COX-2 metabolites.

**Figure 8:**
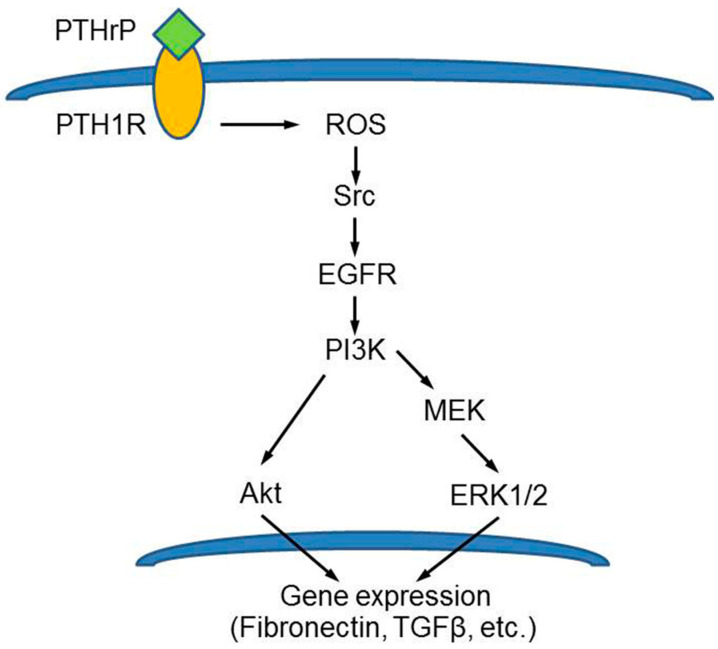
Model depicting the N-terminal PTHrP-induced and ROS-triggered signaling cascade leading to ECM over-deposition in rat mesangial cells. Reprinted from [96]. Distributed under the terms of the Creative Commons Attribution 4.0 (CC BY) license.

In early diabetes, renal hyperfiltration occurs due to an increase in proximal tubule reabsorption. The hyperfiltration state may contribute to structural renal lesions which are preceded by the hypertrophy of the organ. A number of cytokines and molecules are involved in renal changes occurring in diabetes [90,91]. Both PTHrP and PTH1R are abundantly expressed throughout the kidney’s parenchyma, and PTHrP was proven to modulate renal plasma flow and glomerular filtration rate, as well as renal cell proliferation [92]. Experimental evidence suggests that it is involved in the pathophysiology of the diabetic kidney, being upregulated during renal injury [93]. Romero et al. [94] and Ortega et al. [95] investigated the mechanism of high glucose-induced hypertrophy in cultured mouse podocytes, renal tissue explants, and animal models of diabetes, and they found that angiotensin II-induced PTHrP (1–36) overproduction resulted in the increased expression of TGFβ. This, in turn, mediated an early increase in both cyclins D1 and E and cdk2 activity followed by inactivation of cyclin E/cdk, which is an acknowledged molecular signal directing the growth pattern toward hypertrophy. In addition, they also found that p27^Kip1^, inhibitor of the cyclin E/cdk2 complex, was upregulated, thus contributing to the hypertrophy response. The N-terminal PTHrP-stimulated overproduction of p47^phox^, involved in ROS production, was also demonstrated by Chen et al. [96] in rat mesangial cells and linked to the activation of EGFR, Akt, and ERK1/2 signaling, and to the resulting ECM accumulation associated with the fibrotic reaction occurring in the diabetic rat kidney (Figure 8).

## 10. Skin: PTHrP-Related Signatures in Keratinocyte Development and Antiaging Effect

**Figure 9:**
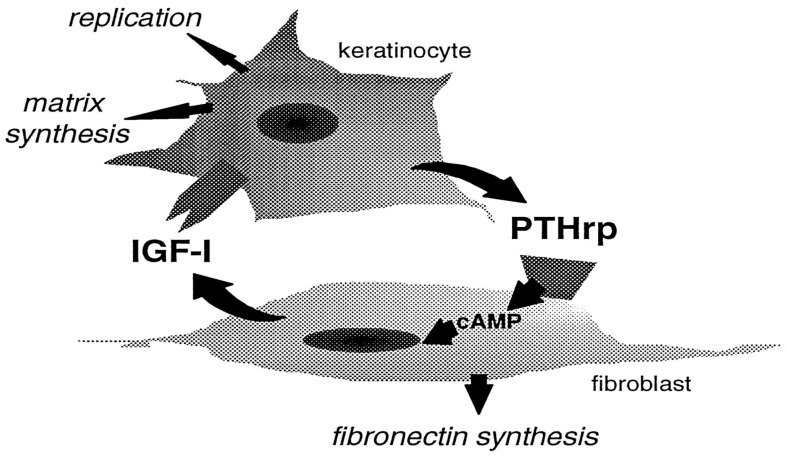
Model depicting the paracrine interactions between dermal fibroblasts and keratinocytes via the PTHrP/IGF-1 circuit. Reprinted from [98]. Distributed under the terms of the Creative Commons Attribution (CC BY) license.

Epidermal keratinocytes are PTH1R-lacking and PTHrP-expressing cells which do not produce but can respond to IGF-1 binding to their surface receptors able to promote their proliferation, as well as ECM protein production [97]. Shin et al. [98] demonstrated the possible paracrine interaction between dermal fibroblasts and keratinocytes in which keratinocyte-derived PTHrP activated cAMP production in PTH1R-exposing dermal fibroblasts and enhanced the PKA-dependent steady state mRNA levels of IGF-1 and its secretion by the latter that, in turn, enhanced keratinocyte development and matrix synthesis, instrumental for skin remodeling and repair (Figure 9).

Keratinocyte growth factor (KGF) is highly expressed by stromal cells from different tissues, including the skin. In contrast, it acts exclusively on epithelial cell types through the KGF receptor (KGFR), stimulating DNA synthesis and supporting the growth of various types of epithelial cells including keratinocytes. To shed further light on the complex epidermis/dermis interplay, Blomme et al. [99] studied the effects of KGF on PTHrP expression and secretion by normal human foreskin keratinocytes (NHFKs) and the effects of PTHrP on KGF expression and secretion by normal human dermal fibroblasts (NHDFs). Their studies demonstrated that N-terminal PTHrP stimulated KGF mRNA and protein expression and secretion by NHDF in a dose-dependent manner, whereas KGF did not regulate PTHrP expression and secretion by NHFK, thereby qualifying the keratinocyte-derived PTHrP as a potential paracrine regulator of KGF expression by dermal fibroblasts in vivo.

Miao et al. [100] generated PTHrP knock-in (PTHrP KI) mice expressing PTHrP (1–84), i.e., a form lacking the NTS and the C-terminus, characterized by the premature aging of the skin. Furthermore, in PTHrP KI mice, they found high levels of the cdk inhibitor P27, whose involvement in the skin differentiation process has long been known [101]. To determine the effects of p27 deficiency on premature skin aging of PTHrP KI mice, Jiang et al. [102] compared the skin phenotypes of PTHrP KI mice to those of p27 knockout mice (p27^−/−^) and of double homozygous p27-deficient PTHrP KI mice (p27^−/−^/PTHrP KI). The P27 shortfall in PTHrP KI mice partially corrected skin premature impairment through a reduction in cellular senescence, as a function of the downregulation of the expression of cdk inhibitors (including p19, p27, and p53) and the upregulation of the expression of cyclin E and CDK2 that the associated molecular signatures evidenced. In addition, ROS accumulation was reduced significantly in p27^−/−^/PTHrP KI mice compared with PTHrP KI mice; therefore, the expression levels of the genes coding for antioxidant enzymes were examined in the skin of the animal models by real-time RT-PCR. Unlike wildtype mice, *SOD1*, *SOD2*, *GPX1*, *GPX4*, *CAT*, and *GSR*, coding for superoxide dismutase-1 and -2, glutathione peroxidase-1 and -4, catalase and glutathione-disulfide reductase, respectively, resulted upregulated in the skin of p27 knockout mice and downregulated significantly in PTHrP KI and p27^−/−^/PTHrP KI mice, thus suggesting that the rescue of the skin-aging phenotype in the latter animals occurred via some degree of recovery of the redox balance.

## 11. Conclusions

Molecular profiling has identified signatures associated with full-length PTHrP or its distinct domains in various differentiated and differentiating model systems in vitro and in vivo, also correlated to the onset of diverse nontumoral and tumoral diseases. In this review, we examined a number of distinct sets of molecular signatures which have been distinguished on the basis of the different histo- and cytotypes considered. These molecular markers are linked to relevant cellular responses and to the activation of transduction pathways, thus expanding the knowledge on the multiple roles of PTHrP in controlling the transcriptional activity and signaling mechanism in a variety of organs with respect to their functional/pathological states. Enhanced understanding of the role of PTHrP in cell fate determination at the molecular level will facilitate new approaches to improve the maintenance of organ homeostasis and assist in developing more effective therapies.

## Figures and Tables

**Table 1:** Variants of PTHrP mRNA produced via alternative splicing at the 5′ and 3′ ends [2].

Promoter	Exons Included at the 5′ End	Ever-Present Exons	Exons Included at the 3′ End Protein Isoform (Amino Acids)
P1	I-II-III	V-VI	VII (139)-VIII (173)-IX (141)
	I-III		
	I		
P2	III	V-VI	VII (139)-VIII (173)-IX (141)
P3	IV	V-VI	VII (139)-VIII (173)-IX (141)

## Data Availability

Not applicable.
